# Creating ‘Local Publics’: Responsibility and Involvement in Decision-Making on Technologies with Local Impacts

**DOI:** 10.1007/s11948-020-00199-0

**Published:** 2020-02-17

**Authors:** Udo Pesch, Nicole M. A. Huijts, Gunter Bombaerts, Neelke Doorn, Agnieszka Hunka

**Affiliations:** 1 Department of Values, Technology and Innovation, Faculty of Technology, Policy and Management, Delft University of Technology, Jaffalaan 5, 2628 BX Delft, The Netherlands; 2 Department of Industrial Engineering and Innovation Sciences, Section for Human-Technology-Interaction, Eindhoven University of Technology, Eindhoven, The Netherlands; 3 Department of Industrial Engineering and Innovation Sciences, Section of Philosophy and Ethics, Eindhoven University of Technology, Eindhoven, The Netherlands; 4 Division ICT – RISE Viktoria, Sustainable Business, RISE Research Institute of Sweden, Gothenburg, Sweden

**Keywords:** Responsibility, Technology appraisal, Expertise, Local democracy

## Abstract

This paper makes a conceptual inquiry into the notion of ‘publics’, and forwards an understanding of this notion that allows more responsible forms of decision-making with regards to technologies that have localized impacts, such as wind parks, hydrogen stations or flood barriers. The outcome of this inquiry is that the acceptability of a decision is to be assessed by a plurality of ‘publics’, including that of a local community. Even though a plurality of ‘publics’ might create competing normative demands, its acknowledgment is necessary to withstand the monopolization of the process of technology appraisal. The paper presents four ways in which such an appropriation of publicness takes place. The creation of dedicated ‘local publics’, in contrast, helps to overcome these problems and allows for more responsible forms of decision-making. We describe ‘local publics’ as those in which stakeholders from the different publics that are related to the process of technology implementation are brought together, and in which concerns and issues from these publics are deliberated upon. The paper will present eight conditions for increasing the effectiveness of such ‘local publics’.

## Introduction

In many cases, the implementation of new technological systems that are thought to benefit society as a whole lead to local forms of opposition because of their possible impact on the local social and natural environment, such as safety risks, environmental harm, affected quality of life, visual intrusion, loss of property value, and privacy. Examples of such resistance can be found in cases like shale gas extraction (Dignum et al. 2015), hydrogen fuel stations (Huijts et al. 2013), carbon capture and storage (Cuppen et al. 2015; Pesch et al. 2017a, b), gas extraction (Mouter et al. 2018), the construction and implementation of flood protection measures (Pigmans et al. 2017), the construction of large wind turbines (Devine-Wright 2005), biofuel production sites (Vochozka et al. 2017), waste landfills (Che et al. 2013), smart LED lighting (Janssens et al. 2020) and ‘smart’ ICT-infrastructures (Sadowski and Pasquale 2015). The impact of technologies on a specific location mobilizes local communities to appeal for their interests and viewpoints to be taken into consideration (Shaw et al. 2015), and even less expected technologies, such as fusion energy, with its own locality concerns (Bombaerts and Laes 2007). The acceptability of a new technology for a local community has come to figure prominently as an essential prerequisite for decisions to be considered legitimate (Van Summeren et al. 2020; Wüstenhagen et al. 2007).

This paper will target the community as a specific sort of ‘public’ that can be set on par with other societal contexts that figure as the ‘publics’ that are to decide upon the acceptability of the new technology. It is important to note here that ‘publics’ are not seen as gatherings of people, but as contexts that elicit responsible forms of behavior. This view follows John Dewey’s (1927) account of responsibility which pertains to the capacity of persons *to respond* to a certain ‘public’ if questions are asked about the moral grounds that motivated their actions. The core problem addressed in this paper is how this capacity of holding persons accountable for their actions is compromised because of the simultaneous presence of plural ‘publics’ that may come with different moral demands and knowledge claims. In other words, there are different ‘publics’ at play, of which no specific group of actors can be designated as *the* ‘public’ that is the legitimate owner or interpreter of the values and interests that have to be considered. It also needs to be emphasized that technology by definition deals with a plurality of ‘publics’, as will be shown in section two, and that it always comes with epistemic and hermeneutic uncertainties that cannot be accounted for by its developers beforehand (Silvast et al. forthcoming). By making a connection between responsibility and the role of ‘publics’ in the assessment of technology, this paper contributes to the expanding literature in the field of responsible innovation (Stilgoe et al. 2013; Cuppen et al. 2019b), which aims to make processes of technology development more responsive to social needs.

The account of the community as one among several ‘publics’ differs from the conceptualization of the ‘public’ as the aggregation of inhabitants of a certain region or as the aggregation of people affected by the implementation of a new technology (e.g. Huijts et al. 2019). Instead, the point of view presented here gives rise to an account in which a plurality of ‘publics’ is dialectically related to the ‘social imaginary’ of a singular ‘public’ (Taylor 2002). In this, use will be made of the notion of ‘counterpublics’ (Fraser 1990; Warner 2002), which has been introduced to denote minority groups that have strived for political recognition. The formation and involvement of counterpublics is necessary to warrant the democratic legitimacy of techno-scientific knowledge in political decisions (also see Callon et al. 2009). Local communities often appear to take the role of a counterpublic, as they formulate contrasting evaluations of the desirability of a certain technology (Pesch et al. 2017a, b).

The paper introduces the notion of a ‘local public’ as a dedicated public that brings together actors from the different publics and counterpublics that are relevant to the decision to be made. With that, a situated and contextualized forum is created that is able to take account of the competing moral demands and knowledge claims that can be connected to the decision-making process about the implementation of the technology. This paper argues that the establishment of such a ‘local public’ would make the process of technology appraisal more responsive than current templates for managing these projects. At the same time, the creation of such an issue-depending ‘local public’ cannot be seen as a sufficient criterion for responsible innovation, but it serves the goal of bringing the community on par with other ‘publics’, so that the overall decision can be said to account for the wider range of ‘publics’ in a symmetrical way, that is to say that the discussion has been free from the exercise of power.

### Outline of the Paper

The next section will describe the theoretical framework concerning the relation between responsibility and different ‘publics’. This will be done on the basis of a philosophical elaboration of the connection between the notions of the ‘public’ and responsibility. The subsequent sections will sketch out four prevalent practical and ideological factors that contribute to misconceptions, each of them affecting the capacity to have responsible forms of decision-making in the context of technologies that have local impacts. Here, the analysis is based on a broad range of literature that deals with cases of technology implementation in local contexts. The following factors will be identified. First, there is the reproduction of dominant perspectives by actors from different institutional backgrounds, who neglect the input of actors that do not represent existing institutional bodies (“The Institutional Reproduction of Dominant Perspectives” section). Second, there is the disavowal of knowledge claims brought forward by lay members of the ‘public’ (“The Disavowal of Lay Knowledge” section). Third, there is the misconception that technology develops autonomously, so that the only choice that a community can make is to either say yes or no, reinforcing the tendency for negative democracy (“The Misconception of Autonomous Technology Development” section). Fourth, there is the pretense of certain actors and organizations to be the ‘real’ representatives of the ‘public’, suggesting that they have a monopoly over what is to be found right or wrong (“The Erroneous Pretense of Representing the ‘Real’ Public” section). Section “Innovating Responsibly: Creating a ‘Local Public’” will present recommendations on how to organize processes of involving ‘publics’ in technology appraisal, by bringing a heterogeneous set of ‘publics’ together into a newly formed ‘local public’ that is contingent on a specific case at hand. The concluding section of this paper (“Final Reflections” section) will discuss and qualify the findings from this study, giving rise to suggestions for further empirical and theoretical research.

## The Moral Autonomy of the ‘Public’ and the Organization of Responsibility

The ‘public’ is a term that is used for many different things; most notably, it connects to things that have an open and/or a collective nature (Weintraub 1997). If used to denote a group of actors, it may refer to an audience, to a government, or to ordinary people (Warner 2002). In this paper, the starting point is the ideological connotations of the concept that follow Charles Taylor’s description of the ‘public’ as a ‘social imaginary’ in which people “imagine their social existence, how they fit together with others, how things go on between them and their fellows, the expectations which are normally met, and the deeper normative notions and images which underlie these expectations” (Taylor 2002, p. 106).

Having the characteristics of a social imaginary turns the ‘public’ into an entity that is fundamentally intangible, it allows the members of civil society to identify with a greater collective that is able to create and preserve a shared social identity (Pesch 2019). In a democracy, this imaginary ‘public’ has to be able to decide its own collective course by engaging in deliberation (Habermas 1985) and it has be able to define its identity independently from external discourses, including expert-based ones (Bombaerts and Laes 2007).

To maintain this normative ideal, the exercise of power by institutions is contained by making actors from these institutions accountable for their actions to the ‘public’. This is organized by having several institutional domains that each has its own ‘public’ that is to be responded to: the state, market and science are structured in such a way that they are responsive to different institutionalized versions of the ‘public’. These ‘institutional publics’ have a clearly identifiable empirical manifestation in the form of the electorate, consumers and scientific peers (Van Gunsteren 1994). Such ‘publics’ can be seen as different *audiences* that look at the activities performed by actors from the institutional realms as spectators do, continuously assessing the desirability of such performances (cf. Green 2010; Lezaun and Soneryd 2007). The scrutiny that is exercised by these ‘institutional publics’ constitutes the possibility of the *ex post* assessment of the legitimacy of an agent’s actions and decisions (Pesch 2005, 2015a).

The depiction of the institutional domains of state, market and science is a crude one, not providing the leverage needed for empirical analyses. However, this tripartition of domains figures very strongly in the way people imagine themselves to be members of a ‘public’ that assesses the actions and decisions taken by actors connected to these institutional domains. As such, this account of responsibility shapes the predominant considerations about how to allocate responsibility within society. At the same time, the efficacy of such ‘publics’ cannot be taken for granted. Political and ideological struggles over the question who may legitimately represent the ‘public’ are as old as the imaginary of the ‘public’ itself. There are also challenges of a more recent date. Institutional domains are increasingly turning into a seamless network, with the integration of science-based expertise in political decision-making, the privatization of public services, industry-university interaction or citizen science. These challenges do not mean that there is no more conceptual validity of the categories of state, market and science, in fact, it is typically the case that such hybrid forms need to be described by making use of these, and related, categories. Moreover, as has been stated above, the concepts of state, market and science should not be primarily seen as empirical descriptions, but as ideal-typical categorizations that allow the construction of ‘publics’ as imaginaries.

The main challenge of crossing institutional boundaries is that it gives rise to incommensurable responsibility demands that evolve from the domains of science, market and politics, as different ‘publics’ are implicated at the same time. Especially the activity of technology development can be seen as an activity that is susceptible for such boundary crossing as it combines knowledge, societal well-being and commercial interests (Pesch 2015a). As such, the assessment of innovation is an activity that is, like the uptake of expertise in policy-making, fundamentally hybrid, recruiting several ‘institutional publics’ at once. Such hybridity gives rise to normative ambiguity and questions about which ‘institutional public’ is the ‘right’ one.

The seamless network of institutional domains creates a singular sphere of authority that goes to the extent of its capacity to be subject to the scrutiny of ‘publics’ (Pesch 2014). With that, the functioning of ‘institutional publics’ as a proxy for the ‘public-at-large’ appears to lose its legitimacy. Instead, social actors as citizens, local communities, and protest movements organize themselves into a variety of counterpublics, making it less clear who constitute the ‘public’ and what the role is of disparate ‘publics’ *vis*-*à*-*vis* authority in decision-making.

## The Institutional Reproduction of Dominant Perspectives

Empirical studies have shown that citizens evaluate the local placing of a technology differently than the placing of the technology elsewhere (Midden and Huijts 2009; Terwel and Daamen 2012; Bombaerts et al. 2020), for instance because of the attachment that people have to the place they live (Devine-Wright and Howes 2010). Moreover, knowing about the local context in which a technology is implemented, people are able to reflect on its concrete and immediate repercussions. The voice of the local population appears not to be taken into account though, as context-specific considerations usually do not fit dominant frames of decision-makers (Cuppen et al. 2015).

This misfit does not evolve out of antidemocratic or otherwise authoritarian inclinations of decision-makers, but usually a *decontextualized conceptualization* of the ‘public’ seems to be used in order to arrive at a decision that seems legitimate (cf. Krzywoszynska et al. 2018). In other words, decision-makers are responding to procedural and legalist expressions of the ‘public’, as the public interest is believed to be embodied by the general legislation and policy (Schubert 1960). It needs to be stressed, though, that such a decontextualized orientation to the ‘public’ does not automatically create a bulwark against contingency and arbitrariness, because, in the end, every concrete operationalization of the ‘public’ can only be a proxy as there is no way to materialize an imaginary. Conversely, the appeal to a given spatial context by a local ‘public’ does not mean that there are no imaginaries involved. Neither version of the ‘public’ is fully real and neither is fully ideal (cf. Walker et al. 2010; Barnett et al. 2012).

In practice, legal and procedural expressions of the ‘public’ typically identify this ‘public’ with a national population, that has given its mandate to governmental activity by parliamentary vote. As such, national jurisdictions tend to override lower jurisdictions (Cuppen et al. 2019a). Decision-makers may also see a local community as an integral part of the national population and not as a distinct ‘public’ out of considerations of procedural justice (Pesch et al. 2017a, b). At least to the perception of the actors involved, the democratic legitimacy of this mutual frame appears to be warranted by the way these actors represent the interests of society by their organizations and by the way that existing legal procedures are followed up (Howard 2015). However, local actors might not have the resource base that is necessary to engage in existing networks of representation, and as such they are prone for being excluded from decision-making processes even if these pertain to them. So, while a neutral and objective stance is then believed to secure fairness, it may lead to the disregarding of the identity of a community (Stewart et al. 2004), and of specific local concerns—this, in turn, ironically contributes to the experience of unfairness among local residents.

There are not only democratic problems with such exclusion; protests of local groups might give rise to previously unarticulated interests, values and viewpoints. In other words, local actors may bring new arguments to the stage that are not part of the dominant repertoire of arguments and as such they are not guarded by any institutionalized stakeholder (cf. Callon et al. 2009; Pigmans et al. 2019). Maintaining a formalistic and legalistic approach increases the chances of marginalizing these new arguments. This problem is especially relevant in case of the implementation of a technological innovation, as this is by definition a new and dynamic phenomenon that cannot be covered by existing assessment frameworks.

Moreover, a legalistic and proceduralist approach imposes the conditions for what is to be considered a legitimate ‘public’ claim or not. If claims or arguments are not in line with the goal-rational frame, authorities tend to not accept them as legitimate forms of the ‘public’ interest (e.g. see Van Asselt and Vos (2008) on the European debate on GMO regulation). The clearest cases of rejecting the legitimacy of public claims take place when local protests are blamed for following a NIMBY-strategy; with that label, such protests are directly stigmatized as being irrational, ill-informed and self-interested (Wolsink 2006). However, the reason for emphasizing the non-universalistic character of unique places does not have to evolve from a lack of rationality, knowledge or morality, but may also come from an alternative conception of the value of place (Drenthen 2010). Moreover, empirical studies have shown that objectors to a technology implementation can be quite well informed (cf. Ellis et al. 2007, p. 520) and that people’s objections are also strongly determined by moral considerations, rather than just based on personal gains and losses (Wolsink 2007). Not only does this repudiation attest to a too exclusive account of what are considered legitimate public claims, it usually leads to even stronger opposition as people feel that their democratic right to express themselves is denied, causing more frustration and distrust (Walker et al. 2011), which in turn can ignite protests that lead to costly delays in implementation, or the complete abandonment of the implementation of new technologies (Dignum et al. 2015). Another reaction of local actors is to copy the form and the content of the dominant discourse in order to increase the chance of being heard by the relevant decision making bodies (Bröer 2008). Though successful perhaps with regards to gaining political leverage, such adaptive strategies challenge the autonomy of a local community to independently decide its own collective course and as such it is hard to see them as desirable.

## The Disavowal of Lay Knowledge

The reproduction of dominant perspectives in the public appraisal of technology mainly pertains to political forms of decision making. An analogous problem may be observed in the realm of knowledge production, which is often exclusively identified with the institutional domain of science. In fact, this problem is strongly related to the reproduction of dominant perspectives, as these dominant perspectives often converge upon the basis of expert knowledge (Majone 1989; Maranta et al. 2003). Still, this point needs separate attention, as such knowledge plays a constitutive role in controversies about the implementation of new technologies (McCormick 2007). By definition, implementation takes place in existing physical, institutional, and social contexts that are fundamentally local, whereas experts may not be able to grasp the complete local character of these contexts (Macfarlane 2003). At the same time, local actors often have specific knowledge about these contexts, which is not always reflected in the knowledge produced by experts (Kerr et al. 2007). The validity of such forms of local knowledge has been demonstrated in the classic study of Wynne (1992) about Cumbrian sheep farmers’ responses to scientific advice about the restrictions introduced after the Chernobyl radioactive fallout. His study shows that laypeople are capable of reflecting upon the epistemological status of their own ‘local’ knowledge in relation to ‘outside’ knowledge. Moreover, new technologies involve uncertainties and complexities that in many cases are underplayed in expert assessments (Doorn and Hansson 2011). In sum, it seems that the ‘public’ that is constituted by experts is not automatically equipped to appraise the impact of technologies, which is why the ‘public’ that is to appraise technology has to be extended by also including societal stakeholders (cf. Ravetz 1996).

As said above, in many ways the disavowal of public knowledge is subjected to the same fallacies as observed in the reproduction of dominant perspectives—hardly surprising, as experts are almost by definition actors that are institutionally embedded (Wynne 2008). Also, experts tend to derive their authority from the way they frame their knowledge, instead of focusing on the content of this knowledge (Bergmans et al. 2015; Irwin 2006). Authority then comes to depend more on the format in which knowledge is presented than on the relevance of this knowledge. The authoritative framing of expert knowledge relies on the articulation of knowledge claims as decontextualized knowledge, meaning that these claims are framed in a universalistic, disengaged, factual manner, separating technology from society, while any other mode in which knowledge claims are presented is discarded as unreasonable (Jasanoff 2003). This way of presenting knowledge claims suggests that experts have access to knowledge that is ‘more true’ than knowledge that is based on situated experience. Most typically, the discarding of situated knowledge neglects the validity of arguments that are presented in an emotional way, while such emotions may reveal moral considerations that have not been taken up by the dominant templates of expertise (Roeser and Pesch 2016). Moreover, it fails to take into account that lay people are very well capable of producing and processing accurate knowledge. After all, the general level of education of the population has been ever increasing over the years, and also, with the rise of the Internet, the possibility of information-gathering and information-sharing has increased immensely. During the process of decision-making on the implementation of a new technology, local actors are often given ample opportunity to turn themselves into experts, not only on the technology at stake, but especially on the implications of the technology in the specific local context (Bombaerts 2004).

## The Misconception of Autonomous Technology Development

The previous two sections presented problems related to sociopolitical processes by which institutional actors secured their authority—even though this may be done unintentionally. The problem that is addressed in this section evolves from the fact that technology development is often misunderstood (Shove 1998). Typically, two conceptions prevail: the first is that the development of a new technology is the mere application of fundamental science (cf. Bijker 1995), the second is that the implementation of technology is a market-based activity (cf. Felt et al. 2013). As an activity that has one leg in the independent realm of science technology and the other leg in the equally independent realm of the market, technology is seen as intrinsically autonomous: it *cannot* be influenced during its development stage and it *may* not be influenced during its implementation stage. Both conceptions are based on the faulty assumption that the processes of technology development and implementation can be described in a linear fashion, while in reality there are many feedback loops between these two processes. In practice, it may be so that patterns are observed that are fundamentally non-linear (Stirling 2010). In other cases, the design of technologies may be adapted after the technology has already been implemented (Bijker et al. 2012). Moreover, the actual development of new technologies takes place in networks of collaborators from science, industry and the market, making modern technology a specialized, multi-actor undertaking (Swierstra and Jelsma 2006). These networks exercise power and as such facilitate or create resistance to and resilience against technology developments (Geels 2014).

As such, it would be more appropriate to say that technology development spans the institutional complex, not only the market, and as such it can be denoted as ‘hybrid’ in its essence (Gibbons 2000). Instead of taking the linear approach to technology development and implementation, it would be better to see the process of innovation as part of a sociotechnical system in which society and technology are in continuous interaction, making the outcome of the innovation process a reiterative and unpredictable process (Pesch 2015b).

Because this systemic character is not sufficiently recognized, acceptability of a new technology is often framed in terms of ‘yes’ and ‘no’ instead of framing it in terms of the conditions under which a new technology is acceptable (Doorn and Fahlquist 2010). In other words, only the *implementation* of a technology is seen as subject to public appraisal, *not the development* of this technology (Hansson 2006). This framing is also recognizable in studies on public acceptance that commonly ask respondents to evaluate a technology as a singular and fixed entity or that only address one particular manifestation of the technology (cf. Huijts et al. 2012; Turcanu et al. 2007), bypassing the dynamics of innovation trajectories (cf. Dosi and Nelson 1994). Having said that, even in the appraisal of the implementation of a technology, the capacity for public appraisal is often disputed, because it conflicts with the idea of a free market in which the ‘public’ decides the acceptability of a new technology by not purchasing or using it, disavowing the hybrid nature of innovation processes. However, public appraisal is not reserved for the ‘public’ of the market, but pertains to all relevant ‘publics’, also those associated with society, science and politics.

Technology appraisal should not primarily give a decisive answer about whether a technology is to be implemented or not, but should give the conditions under which it is acceptable that the technology is implemented (Pesch and Ishmaev 2019). Ever since Langdon Winner’s classic study on the low overpasses of Long Island that were designed to prevent poor and black people from visiting the beaches by bus (Winner 1980), it has become widely accepted that values and ideologies may be directly designed into technologies—taking away their value-neutral pretext. As such, the development of the technology, including its implementation, should be subjected to redesign so that a wider set of values are included (Taebi et al. 2014). Likewise, the study of public acceptability should not be based on the assumption that a technology is an end-product that can only be the subject either of consent or of refutation as an unchangeable unity.

## The Erroneous Pretense of Representing the ‘Real’ Public

The three problems that have been identified until now have in common that they relate to the monopolization of a ‘public’ that is associated with a particular institutional domain. This leads to unresponsive forms of innovation as technology development includes a range of different ‘publics’ including categories such as the ‘general public’ and ‘local communities’. One of the main claims here is that these ‘publics’ need to be part of the process of appraisal. At the same time, one should also be aware that other parties that claim to act as the legitimate representatives of the imaginary of the ‘public’ lack the right to do so.

Not only the ‘technocratic pitfall’ that leads to the exclusion of arguments and sentiments of local populations should be avoided, but also the ‘populist pitfall’ by ignoring the complexity of values, knowledge and interests at stake and by presupposing that only voices from outside the institutional system are credible expressions of what the ‘public’ really wants (cf. Roeser 2011). Populist voices have no more epistemic or democratic legitimacy than expert voices (cf. Durant 2011), and the values and concerns that are forwarded by these voices should be considered seriously in decision-making processes, but they should not be given a special status. In the end, a wide range of different knowledge claims and moral demands have to be deliberated upon so as to arrive at a trade-off that receives broad consent. Populist voices should be considered as attempts to adjust institutionally embedded articulations of the ‘public-at-large’, which are presented by counterpublics that emerge in reaction to discontent with dominant frames and assessments (cf. Callon and Rabeharisoa 2008). Populist expressions of particular issues can be seen as a form of learning (Cuppen 2018), in the sense that new perspectives, values, or knowledge claims are added to existing repertoires, so that the decision-making process is enriched.

At the same time, the social dynamics that are typical to populist activity might make it hard to maintain such a constructive approach. The legitimacy of populist movements and their spokesmen often depends on their status as outsiders to prevalent institutional frameworks. Not only does this mean that populist actors may be reluctant to join a decision-making process, consent with the outcomes of such a process may also be unlikely. Moreover, populist voices do not have to be expressed by members of the local community, making it hard to assess their validity as expressions of a certain ‘public’.

## Innovating Responsibly: Creating a ‘Local Public’

The previous sections presented four ways to monopolize the claim for publicness. Each of them stands at odds with the need for having community acceptability of technologies, as they take away the capacity to seriously attend to the knowledge claims and moral demands of local communities and as they obstruct meaningful patterns of communication between different ‘publics’. An underlying problem here is that ‘one-size-fits-all’ institutional patterns are reproduced without any reflexivity and openness towards the emergence of new interests, knowledge claims, values and preferences.

In case of a specific project, a way to circumvent this problem is to see the process of technology appraisal as a process that is local, contextual, and conditional, revolving around a direct and concrete problem or activity and leading to specific and detailed arrangements, which are agreed upon by those actors involved and that only pertain to the practice at stake (Pesch 2014). This does not at all mean that a technology has no impact on wider geographical scales or that a local public should be seen in isolation (cf. Chilvers and Longhurst 2016), but in order to assess the acceptability for a ‘local public’, the appraisal should pertain to this situated character, instead of a decontextualized understanding of the technology. With the starting point of a ‘public’ that is connected to such a dedicated process of appraisal, local actors can actively engage themselves in the decision-making process, by attending to the specific issues that these actors relate to as a collective (cf. Krabbenborg 2016; Marres 2007). As such the process of technology appraisal will be informed by the shared understanding of the members of a local community and vice versa (Zaal et al. 2014).

The confrontation of a local community with a new technology basically creates a new ‘public’ that has new issues and concerns, which should be included in the decision-making process with the aim to embed its values, interests and concerns in the technological design (cf. Pellizzoni 2003; Pesch et al. 2017a, b). The introduction of a dedicated ‘local public’ is believed to accommodate the issues of local actors because it puts these on par with the values and interests that are embedded in existing institutional procedures and arrangements by forming an assembly of stakeholders that represents those ‘publics’ that are relevant with regards to the given project on technology implementation. To align with the hybrid nature of decision-making on technology implementation, such a ‘local public’ should act as a ‘hybrid forum’: a space that is open for heterogeneous groups to “come together to discuss technical options involving the collective” (Callon et al. 2009, p. 18). This ‘local public’ has a temporal character, bringing together different ‘publics’ in a setting that is goal- and context-specific. It allows the appraisal of the technology to be made in a way that is responsible to a ‘public’ that may be conditional, but at least has an empirical quality.

Below, a number of conditions will be sketched out that need to be attended to while organizing a ‘local public’ designated to assess the acceptability of the local implementation of a new technology. Without claiming to be exhaustive or novel, eight conditions will be given that help to increase the effectiveness and legitimacy of such ‘local publics’. In many respects, these conditions will build on existing participatory methods that have been developed ensuing the so-called ‘deliberative turn’ in policy studies (Fischer 1999; Mohr 2011). At the same time, this approach explicitly looks at the way that assessments and decisions may be connected to specific responsibility arrangements, so as to do justice to the contrastive ideological and practical demands that are intrinsic to processes of democratic decision-making (Bohman 1998).

### Condition 1: A Symmetrical Selection of Actors

A selection of actors from the different ‘publics’ at hand should be made, including local actors, institutional actors, and experts who are to be engaged in a dialogical process so to articulate the values, interests and concerns that are relevant with regards to the technology that is projected to be implemented. Such a ‘local public’ should fulfill the conditions that allow the participation of local actors, institutional actors, and experts in a symmetrical fashion using their own strengths in the debate (cf. Roeser and Pesch 2016). In this, ‘symmetrical’ refers to a ‘power-free’ dialogue, in the sense that no actor has authority over another based on a given position or function (Habermas 1985)

### Condition 2: A Case-Specific Approach

For particular projects, the selection of participants has to be made on a case-specific basis, as the range of local actors affected by a new technology depends on the geographical, political and institutional boundaries that are at stake in a given project. No pre-determined criteria can be given here, as such boundaries are usually contingent on the nature and development of such cases. At the same time, it needs to be acknowledged that any demarcation line is artificial, forwarding a binary decision about who is considered to be affected by the project, and who is not. Such decisions are often based on regulatory boundaries or on technical thresholds, for instance related to the distance from a certain project or the measurable impact in visual or acoustic terms. While necessary to allow for decision making, such a binary demarcation does not align well with the nature of technology-based projects. Firstly, the level of nuisance caused by such projects can be better characterized by using a continuous than a discrete scale. Secondly, the use of a singular principle reduces the scope of impacts of a project, while there may be a heterogeneous array of impacts that, furthermore, cannot always be expressed in measurable terms. Thirdly, technologies often have impacts on multiple geographical scales, as they frequently entail networks of interdependent technologies. These considerations prompt the organizers of a local public not to take the boundaries of that public to be too narrow and to be flexible with regards to new concerns and insights. This means, as will be reiterated at the end of this paper, that any chosen demarcation should be open for renegotiation.

### Condition 3: The Need for Political Leverage

It must be clear from the start of the process that the ‘local public’ has political leverage. The results of the participatory process should be genuinely taken into account in the actual decisions themselves. A ‘local public’ without such a negotiable position is not only too weak in the power play, it also loses its legitimacy and its appeal (Bogner 2012).

### Condition 4: A Level-Playing Field

Interactions taking place in a ‘local public’ should be based on a dialogical form of deliberation: no party should prescribe the rules of the game in terms of which interests are valid, which discursive frames are valid, and which modes of expression are acceptable (cf. Jami and Walsh 2017). A level playing field should be created among all actors involved; all actors have to be able to forward knowledge claims about the impacts of the technology and their local concerns, including when these are emotionally charged (Roeser 2012). This condition might lead to a reluctance of parties to partake in the process, as stated above, since populist movements rely on their outsider status. In such cases, a pragmatic approach has to be followed for identifying moral demands and knowledge claims that are endorsed by these parties, while not brought to the table of the ‘local public’, for instance by inviting other spokesmen for these demands and claims.

### Condition 5: An Ex Ante Agreement on the Rules of the Game

A level playing field cannot be created if the involved actors do not agree beforehand on the rules of the game—comparable with, for example, Rawls’ idea of a wide reflective equilibrium (Doorn 2010, 2013; Rawls 1974) or other models that stand in the tradition of procedural political theory (e.g. Einsiedel et al. 2001). These rules have to include normative diversity as a starting point, acknowledging the fact that the quality of a democratic process depends on its capacity to take a plurality of viewpoints on board (Cuppen et al. 2019a).

### Condition 6: Ex Post Assessment of the Validity of Claims

The rules of the game should include that the legitimacy of the statements that are brought forwards during this dialogue should only be assessed *after* they are introduced. Claims cannot be authorized upon the basis of existing institutional roles, but have to be assessed from the perspective of the ‘local public’ at hand (Durant 2011)—a condition that is usually not attended to in literature on participatory methods. One particular set of rules is very important. Knowledge and normative statements should be treated symmetrically. This means, for instance, that lay knowledge is not excluded beforehand, but may be put forward and subsequently its scientific legitimacy should be assessed as a knowledge claim—for instance by demanding review by independent experts that are trusted by the full ‘local public’. Given the condition for symmetry, it is also expected that lay actors accept the outcome of such an assessment. A similar point could be made about normative claims referring to particular values or ideological perspectives. In the end, the process of appraisal should not neglect its ultimate goal which is to design the technology in line with the discussed values, not merely to accept or refute it (Correljé et al. 2015)—which, it has to be stressed, does not exclude the option of refuting the implementation of the technology full stop.

### Condition 7: Dealing with Group Dynamics

Internal group dynamics play an important role in any participatory setting and need to be guarded. There are differences in attitude and personality, with regards to traits such as assertiveness, intelligence, or expressiveness (cf. Cuppen et al. 2009). Participatory methods of decision-making are often subjected to strategies and power play, and participatory settings are used as venues for seeking political gain—there is no reason to be naive in these matters (Bogner 2012). Participatory settings may also give rise to ‘group think’ or may be methodologically inclined to seek consensus (Huitema et al. 2007), leading to agreement among the participants so that their original interests and viewpoints may become compromised (in the literal sense of the word), decreasing the representativeness of the whole enterprise.

### Condition 8: Keeping Track of the Process

As stated above, there are no pre-given criteria to select participants of the ‘local public’. Moreover, the internal group dynamics may affect the functioning of the ‘local public’. This means that a sharp eye has to be kept on the process. It demands that the organizers and/or moderators of the process continuously monitor the progress of the process by attending to questions like: is the ‘local public’ still representative?; is the symmetry between participants respected?; are the rules complied with?; and so on. Not only do the organizers and moderators keep track of the progress, they also have to intervene if it adopts an undesirable trajectory. In doing so, it will help if the assembled ‘local public’ is not the only source of information. A ‘local public’ is a form of an ‘invited public’ and cannot be seen as representative of the population and its concerns (Wynne 2007; Cuppen 2018). To get insights into the points of view of those actors who are ‘uninvited’ and other actors who are not involved, methods such as surveys among the community may figure as a check for whether their representatives can still be regarded as genuine representatives (Pesch 2019). Although the use of surveys may often be seen as opposing the demands of deliberative democracy (e.g. Callon et al. 2009, p. 156), they can still serve as an additional validation of claims made by the assembled ‘local public’ (cf. Lezaun and Soneryd 2007). Obviously, such surveys also have to comply with the terms as sketched out in this paper: the technology should not be presented as a yes-or-no question, but as work-in-progress. Also, diverse types of ethical evaluations and stakeholder analyses will be helpful to give a more comprehensive understanding of the concerns of a ‘local public’ that could be taken into consideration (cf. Roeser and Pesch 2016).

## Final Reflections

The driving hypothesis of this paper is that the construction of a ‘local public’ serves decision-making processes on technologies that have local impacts by increasing the responsiveness to the demands of the heterogeneity of ‘publics’ that are relevant to these decisions. The analyses presented here have shown the tendencies of actors to strategically connect the label of publicness to their own normative and intellectual outlook, which undermines the legitimacy and eventually also the effectiveness of the decision-making process.

The establishment of a case-specific ‘local public’ is believed to overcome these problems. It builds on existing literature on participatory assessment of technology, but it also adds a number of considerations. Most notably, it articulates these issues in terms of responsibility to a heterogeneity of publics and with that overcomes the tendency in the literature to assume binary oppositions between a public that is either autonomous or ignorant and systemic institutions that are either technocratic or authoritative. As such, this account offers a more productive approach that allows one to put the different ‘publics’ on par, as well as allowing for a more comprehensive form of assessment.

At the same time, it needs to be admitted that the hypothesis about the efficacy of ‘local publics’ is primarily based on theoretical considerations and its operational ramifications need to be researched in practical applications. To start with, it is still very much an open question as to what extent actors are willing to cooperate—some actors may have strategic motivations not to join a local public, for they might consider it to compromise their credibility, for instance their status as outsider or as neutral parties. If these starting points are seen as non-negotiable, it might be hard to get such parties to consent to the rules and the outcomes of a participatory decision-making method that follows the conditions sketched out above (cf. Wynne 2007).

A second question evolves from this first point. It may be so that non-involvement does not necessarily have to create fundamental problems, as pragmatic solutions are endorsed that allow the identification of the issues at stake, including values, concerns and insights of non-involved parties. At the same time, this raises questions about which proxies and heuristics work. It is expected that proxies and heuristics needed to be experimented with before anything can be said about their efficacy.

Another set of questions relates to the boundary conditions for a ‘local public’. Where does the demarcation lie between those actors who are ‘in’ and who are ‘out’ and which demands and claims are taken into account or not? These boundaries involve geographical boundaries, but also boundaries of expertise and interest. It is not expected that a pre-given set of principles will allow the determination of such boundaries in a generic way; however, it will be possible to develop a set of heuristic guidelines that will help to determine the boundaries of the different ‘publics’ that need to be included in a ‘local public’ (also see Gehrke 2014). Another issue here is that some ‘publics’ may overlap, in the sense that they share members or have similar interests and outlooks, or in creating related networks of ‘local publics’ (also see Chilvers et al. 2018). Again, heuristic guidelines need to be developed to manage such overlap.

In all, these operational questions invite pragmatic solutions. In the end, a ‘local public’ is subject to the same theoretical problem that has been presented earlier: it is a proxy of a ‘public’ that is imaginary in its essence, and as such it can never be the ‘real thing’ itself. Ideally, the findings of the ‘local public’ should be open to renegotiation itself, so their legitimacy can be adjusted. In all, this very much compels tailor-made approaches, instead of using a one-size-fits-all approach. Having said that, the conditional nature of a ‘local public’ needs to be qualified as well, it will have real-life repercussions: once a technology is implemented, it often becomes impossible to remove or adapt it. In this, the conditions that are proposed here will not guarantee the public acceptability of a new technology, but they are believed to contribute to the overall responsibility and legitimacy of the decision-making process on the implementation of the technology. In the end, being engaged in a ‘local public’ provides learning experiences for public authorities, companies and experts, making them more receptive to values and concerns that emerge in a local context.
